# Sweet attraction: sugarcane pollen-associated volatiles attract gravid *Anopheles arabiensis*

**DOI:** 10.1186/s12936-018-2245-1

**Published:** 2018-02-21

**Authors:** Betelehem Wondwosen, Göran Birgersson, Habte Tekie, Baldwyn Torto, Rickard Ignell, Sharon R. Hill

**Affiliations:** 1 Department of Zoological Sciences, Addis Ababa University, Box 1176, Addis Ababa, Ethiopia; 2 Disease Vector Group, Department of Plant Protection Biology, Swedish University of Agricultural Sciences, Box 102, Sundsvägen 14, 230 53 Alnarp, Sweden; 3 Behavioural and Chemical Ecology Department, International Centre of Insect Physiology and Ecology, P. O. Box 30772, Nairobi, 00100 Kenya

**Keywords:** Mosquitoes, Attraction, Stimulation, Cultivars, Pollen volatiles, Gravid, Oviposition

## Abstract

**Background:**

*Anopheles arabiensis* is a key vector for the transmission of human malaria in sub-Saharan Africa. Over the past 10,000 years, humans have successfully cultivated grasses and altered the landscape, creating *An. arabiensis* favourable environments that contain excellent habitats for both larvae and adults. Sugarcane is the most expanding agricultural system in sub-Saharan Africa, and is linked to the increased threat of malaria in rural communities. The prolific production and wind dispersal of sugarcane pollen, together with standing pools of water, often provide, as a result of irrigation, a nutrient-rich environment for the offspring of gravid malaria mosquitoes.

**Results:**

In the present study, sugarcane pollen-associated volatiles from two cultivars are shown to attract gravid *An. arabiensis* in a still air two-port olfactometer and stimulate egg laying in an oviposition bioassay. Through combined gas chromatography and electroantennographic detection, as well as combined gas chromatography and mass spectrometric analyses, we identified the bioactive volatiles and generated a synthetic blend that reproduced the full behavioural repertoire of gravid mosquitoes in the Y-tube assay. Two subtractive odour blends, when compared with the full blend, were significantly more attractive. These three and four-component subtractive blends share the compounds (*1R*)-(+)-α-pinene, nonanal and benzaldehyde, of which, (*1R*)-(+)-α-pinene and nonanal are found in the attractive odour blends from rice plants and maize pollen. In pairwise comparisons, the rice synthetic odour blend was more attractive to gravid mosquitoes than either of the pollen blends, whereas the pollen blends did not differ in attraction.

**Conclusions:**

The attraction of gravid females to sugarcane pollen volatiles demonstrated in this study, together with the previously found grass-associated volatiles, raise the potential of developing a bioactive chimeric blend to attract gravid malaria mosquitoes. This is discussed in relation to the development of novel and cost-effective vector control measures.

## Background

Stagnant transient water bodies with attendant emergent vegetation are frequently associated with the presence of *Anopheles gambiae* sensu lato (s.l.) malaria mosquito larvae [1–7]. Consequently, such habitats are associated with high mosquito population densities and an increased risk of malaria in surrounding inhabited areas [8–12]. While the natural habitats of *An. gambiae* s.l. larvae are commonly associated with wild grasses of the family Poacae [1–7], other types of vegetation, including papyrus and floating mats, are negatively correlated with larvae presence [1, 13, 14].

Grass-associated habitats have expanded to include agricultural areas with domesticated grasses [10, 15–17]. The domestication, irrigation and continued cultivation of Poaceae grasses, such as maize, rice and sugarcane, provide larval nutrients, e.g. from pollen, that enhance the development and survival of *Anopheles arabiensis* larvae, above and beyond that provided by wild grasses [18, 19]. Such an association between the vector and crop suggests that mosquitoes have selectively adapted to these domesticated grass habitats [1, 10, 20–22], and is reflected in the odour-mediated response of gravid mosquitoes to these crops and their pollen [23, 24].

To increase our understanding of the oviposition ecology of *An. arabiensis*, and to enable the development of novel control strategies for this key vector, we have previously identified multi-component, behaviourally active, odour blends that reproduce the behavioural response of gravid mosquitoes to maize pollen [24] and rice plants [23] under laboratory and semi-field conditions. While these blends share (*1R*)-(+)-α-pinene, (*R*)-(+)-limonene and nonanal, the full behavioural repertoire of gravid mosquitoes is contingent on the presence and natural ratio of all of the identified compounds in the synthetic odour blends [23, 24]. This study expands the understanding of the chemical composition of grass-associated odours involved in the attraction and stimulation of gravid malaria mosquitoes to oviposition sites in the context of the natural environment. As one of the most expanding production systems in sub-Saharan Africa, large scale irrigated sugarcane has been linked to the increased prevalence of malaria through increased vector populations [12, 25]. Similar to maize, sugarcane pollen is prolifically produced [26] and wind dispersed [27], resulting in pollen-rich pools of standing water as potential mosquito oviposition sites and larval habitats. The effect of sugarcane pollen-associated volatiles on oviposition site selection by gravid *An. arabiensis* were investigated and compared with the previous work on maize pollen and rice plants [23, 24]. The potential of creating a “chimeric lure” eliciting an enhanced attraction of gravid malaria vectors is discussed. The combination of a chimeric blend with a trap or contamination station has the potential to be incorporated as a component of integrated vector management for future sustainable management of the vector.

## Methods

### Experimental mosquitoes

The experimental mosquitoes used in the behavioural study were obtained from established laboratory reared colonies of *An. arabiensis* (Mbita strain) at the International Centre of Insect Physiology and Ecology (ICIPE) Duduville campus, Nairobi, Kenya. For the electrophysiological bioassay, *An. arabiensis* (Dongola strain) were reared at the Swedish University of Agricultural Sciences (SLU), Alnarp, Sweden. The aquatic stages were kept in plastic rearing trays filled with distilled water (2.5–5.5 L) to a depth of 4–5 cm. The larvae were provided with TetraMin^®^ fish food (Tetra, Melle, Germany) and the water was changed every other day. Pupae were transferred to cups (0.5–1 L; 4–5 cm depth) in cages (30 cm × 30 cm × 30 cm; custom made or Bugdorm, MegaView Science, Taiwan) until adults emerged. Honey or sucrose solution (10%) was provided ad libitum for adults that were maintained under standard conditions 27 ± 2 °C, 75 ± 5% relative humidity under a 12 h light:12 h dark cycle. Female *An. arabiensis* (5-days post-emergence) were provided with blood (artificial feeder: defibrinated sheep or rat blood; or the arm of a volunteer) once a day for 15–30 min over 2 days. For both the behavioural and electrophysiological bioassays, female *An. arabiensis* were used 3-days post-blood feeding.

### Headspace volatile collections

Headspace volatile collections were made from Coll 48 and EAK 71-402 sugarcane cultivars obtained from Mombasa, Kenya. The volatiles were collected during the day from single, cut, fully mature, male flowers in a closed loop head space system (40 replicates per cultivar). Air (medical grade, Medical Air, BOC Kenya, Nairobi, Kenya; 1 L min-1), passing through copper tubing, was filtered through activated charcoal and humidified by passing through double distilled water and flowed through multiple parallel ports connected to the odour sources enclosed in air-tight glass chambers (2 L) for 5 h. The volatiles from the male flowers were adsorbed onto Super-Q aeration columns (30 mg, mesh 80/100, Analytical Research System, Gainesville, Florida, USA). The aeration columns were made from ca. 6 cm Teflon tubes (ID ~ 3 mm) filled with Super Q, which was kept in the tubes by two stoppers of polypropylene wool. Aerations were each eluted with 600 μL GC/GC–MS-grade dichloromethane (DCM) (Burdick and Jack-son, Muskegon, Michigan, USA) and kept at − 80 °C until used. Control aerations 95 were performed in parallel, without flowers, and the columns eluted with the same batch of DCM.

### The still air two-port olfactometer

The still air two-port olfactometer [23] was used to test the response of gravid *An. arabiensis* to: (1) headspace aeration extracts of each of the cultivars against DCM; (2) headspace aeration extract of Coll 48 against EAK 71-402, and (3) full synthetic blends, based on the GC-EAD and GC–MS analyses of the pooled aeration extract of Coll 48 and EAK 71-402, against DCM. For each experiment, ten gravid females were placed in custom-made cages (22 cm × 30 cm × 12 cm) and allowed to acclimatise for 5 min. The behavioural responses to increasing amounts of the aeration extracts corresponding to volatiles released during 16–80 min from Coll 48 and EAK 71-402 were analysed. Two dental-wick odour dispensers (4 cm × 1 cm; L:d; DAB Dental AB, Upplands Väsby, Sweden) containing either the extracts or control, as described above, were simultaneously introduced into the cylindrical vinyl arms (13 cm × 9 cm; L:d) positioned at opposite ends of the cage, and the ends were covered by mesh. Ten replicates per treatment and per release rate were performed. Synthetic blends were prepared at six different doses, in half orders of magnitude, between 5 and 1650 ng min^−1^ of, (*1R*)-(+)-α-pinene in pentane. The ratio among the compounds in the blend was maintained as a constant across all doses. Then, dose response assays were conducted with the full blend against its solvent control. The mosquitoes that landed in either of the arm were recorded after 5 min and each experiment was replicated ten times. Fresh mosquitoes were used for every replicate. All assays were conducted between 18:00 and 21:00 under 27 ± 2 °C, 75 ± 5% relative humidity conditions.

### The oviposition bioassay

The oviposition bioassay [23] was used to determine the oviposition preference of gravid *An. arabiensis* for the headspace extracts and synthetic blend. Cages (30 cm × 30 cm × 30 cm) covered with white nylon mosquito netting, containing two 100 ml polyester cups (Qingdao Ori-Color Industry and Commerce Co., Ltd., China) filled with distilled water (100 mL) were placed in opposite corners, 4 cm from each wall. The treatment cups were conditioned by dispensing the headspace volatile extracts of Coll-48 and EAK 71-402, or the synthetic blend, directly onto the surface of the water. Release rates of the compounds from the oviposition cups, as well as the integrity of the blend, were analysed as previously described [23]. The control cups were conditioned with equal volumes of DCM. The position of the cups was exchanged between experiments. Ten gravid mosquitoes were transferred from the maintenance cage at dusk (18:00), and the numbers of eggs in the two cups were counted on the following day (09:00). Ten replicates were performed for each experiment and release rate. To assess whether the presence of eggs from other individual females in the oviposition substrate affected subsequent laying behaviour, control (water + DCM) vs. control experiments were performed with both single individuals and in batches of ten. No bias towards either control was observed (***χ***^2^ = 1.002, 95% CI 0.994–1.011; P = 0.563).

### The electrophysiological bioassay

The electrophysiological bioassay recorded the antennal response of gravid mosquitoes to the pooled aeration extract using combined gas chromatography (GC) and electroantennogram detection (EAD) analysis. A pulled glass microcapillary reference electrode, filled with Beadle–Ephrussi Ringer, was inserted through the foramen of the excised head. A similarly prepared recording electrode was connected to the cut distal end of the antenna and attached to a pre-amplifier probe, connected to a high impedance DC amplifier interface box (IDAC-2; Syntech, Kirchgarten, Germany). Then, 2 μL of sample was injected onto the GC in splitless mode (30 s, injector temperature 225 °C). The Agilent Technologies 6890 GC (Santa Clara, CA, USA) was equipped with a HP-5 column (30 m × 0.25 mm i.d, 0.25 μm film thickness, Agilent Technologies), and hydrogen was used as the mobile phase at an average linear flow rate of 45 cm s^−1^. The column oven temperature was programmed from 40 °C (3 min hold) through 10 °C min^−1^ increments to 250 °C (held for 5 min). In the GC effluent, 4 psi of nitrogen was added and split 1:1 in a Gerstel 3D/2 low dead volume four way-cross (Gerstel, Mülheim, Germany) between the flame ionization detector and the EAD. The GC effluent capillary for the EAD passed through a Gerstel ODP-3 transfer line that tracked the GC oven temperature, and into a glass tube (10 cm × 8 mm), where it was mixed with charcoal-filtered, humidified air (1.5 L min^−1^). The antenna was placed 0.5 cm from the outlet of this tube. The antennal responses to the separated pooled headspace volatiles were recorded and analysed using the software GC-EAD 2011 (V.1.2.3, Syntech, Kirchzarten, Germany).

### Chemical identification

Chemical identification of the biologically active compounds recorded through the GC-EAD analysis was accomplished using GC and GC-mass spectrometry (GC–MS; 6890 GC and 5975 MS; Agilent Technologies), operated in the electron impact ionization mode at 70 eV. Samples were analysed on two different fused silica capillary columns (60 m × 0.25 mm, 0.25 μm film thickness), coated with DB-wax (J&W Scientific, Folsom, CA, USA) or HP-5MS (Agilent Technologies) and compared with the control aeration extracts. Helium was used as the mobile phase at an average linear flow rate of 35 cm s^−1^. Two microlitres of the sample were injected. The column oven program was the same as for the GC-EAD analyses, described above. Compounds were identified according to retention times (Kovat’s indices) and mass spectra, in comparison with custom made and NIST05 libraries (Agilent). The identified compounds were confirmed by co-injection of authentic standards: *o*-xylene (CAS no. 95-47-6; Sigma-Aldrich, 98%), styrene (CAS no. 100-42-5; Aldrich, 99.5%), 1,8-cineole (CAS no. 470-82-6; Sigma, 99%), undecane (CAS no. 1120-21-4; Aldrich, 99%), *N*-ethylbenzenamine (CAS no. 103-69-5; Aldrich, 98%), dibutyl phthalate (CAS no. 84-74-2; Sigma, 99%), (*1R*)-(+)-α-pinene (CAS no. 7785-70-8; Sigma, 98%), nonanal (CAS no. 124-19-6; Sigma, 95%), benzaldehyde (CAS no. 100-52-7; Sigma, 99%), *p*-cymene (CAS no. 99-87-6; Aldrich, 97%) and eicosane (CAS no. 112-95-8; Sigma, 99%). Co-injection of an internal standard, 100 ng heptyl acetate (99.8%; Aldrich) was used for quantification. While dibutyl phthalate is a known plasticiser, and *o*-xylene and styrene are common contaminants in DCM, none of these compounds were identified from the control aerations, and subsequently these compounds were included in the behavioural analyses.

### Synthetic blend pairwise comparisons

Synthetic blend pairwise comparisons were performed to assess any difference in the level of attraction of gravid *An. arabiensis* to the behaviourally active blends, at the optimal release rates, identified from maize pollen (10 ng min^−1^) [23], rice plants (10 ng min^−1^) [24] and sugarcane (50 ng min^−1^) in the Y-tube assay [28]. Mosquitoes were flown in the assay in groups of ten, and ten replicates were performed.

### Subtractive blends

Subtractive blends in which single or multiple compounds were removed, were tested for the response of gravid *An. arabiensis* against the full blend using a Y-tube assay [28]. The subtractive blends were released as described above. Subtraction of the compound(s) was compensated for by making up the volume of the missing compounds with the addition of the solvent pentane. Mosquitoes were flown in the assay in groups of ten, and ten replicates were performed.

### Statistical analysis

The statistical analysis of the behavioural responses by gravid *An. arabiensis* in the two-port olfactometer and oviposition bioassays was done using binary logistic regression in SPSS Statistics for Windows, Version 20 (Armonk, NY: IBM Corp). The choice was the dependent variable, weighted by the number of (1) mosquitoes in the attraction assays and (2) eggs laid in the oviposition assays, with release rate or dose as the independent fixed effect and replicate (day) as a random effect. For visualisation, attraction preference (AP) and oviposition preference (OP) indices were calculated using; AP = T − C/T + C and OP = T − C/T + C, where T is the number of mosquitoes or eggs associated with the test odours and C the number of mosquitoes or eggs associated with the solvent controls. The use of the terms “attraction” and “oviposition preference” in this study refer to an increased number of mosquitoes that make source contact within the treatment arm in the olfactometer and an increased number of eggs present in the treatment cup, respectively, compared with the concordant controls.

## Results

### Gravid malaria mosquitoes respond to the odours associated with sugarcane pollen

**Fig. 1:**
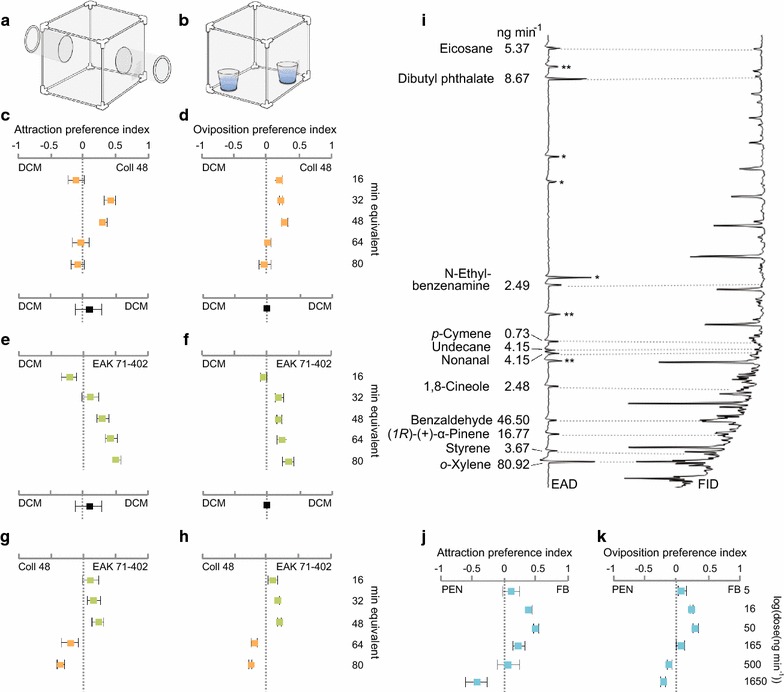
Gravid *Anopheles arabiensis* respond to sugarcane pollen-associated odours. Schematics of the two-port olfactometer (**a**) and oviposition (**b**) assays. Attraction (**c**, **e**) and oviposition (**d**, **f**) preference of mosquitoes to the pollen headspace volatiles of the Coll 48 (light orange) and EAK 71-402 (green) sugarcane cultivars compared to the dichloromethane (DCM) control, respectively. Solvent only controls (DCM vs. DCM) are indicated (black) (**a**–**d**). An attraction or oviposition index of zero indicates a preference for neither treatment nor control. Attraction (**g**) and oviposition stimulation (**h**) of gravid *An. arabiensis* to the headspace volatile extracts of Coll 48 compared to that of EAK 71-402. **i** Electroanntenographic detection (EAD) traces depict voltage changes (mV) in response to the bioactive compounds in the pooled headspace extracts of both sugarcane cultivars, eluting from the gas chromatograph and registered by the flame ionisation detector (FID). Asterisks indicate responses to unidentified aromatic compounds (*) and other compounds present in the control headspace extracts (**). The identity and release rate of the bioactive compounds are shown at the left. A synthetic blend (blue; FB) composed of the eleven bioactive compounds identified, in their natural ratio (**i**), elicited attraction (**j**) and stimulated oviposition (**k**) in gravid mosquitoes in a dose-dependent manner when diluted in pentane (PEN). Ten replicates, of 10 mosquitoes each, were used in each behavioural experiment. Error bars represent standard errors of the mean

To investigate the behavioural response of gravid *An. arabiensis* to the volatiles associated with sugarcane pollen, the headspace extracts of individual sugarcane male flowers containing pollen from two cultivars, Coll 48 and EAK 71-402, were collected and tested in a two-port olfactometer (Fig. 1a) and an oviposition bioassay (Fig. 1b). Gravid mosquitoes showed a dose-dependent attraction and preference to oviposit in response to the headspace extracts of Coll 48 over the solvent DCM alone [Wald, ***χ***^2^ = 0.743, 95% Confidence Interval (CI) 0.561–0.984, P < 0.037; ***χ***^2^ = 0.993, 95% CI 0.990–0.997, P < 0.0001; Fig. 1c, d]. The range of *An. arabiensis* behavioural sensitivity to the headspace of Coll 48 appeared to be constrained, as a loss of attraction and preference was observed at the two highest release rates tested 191 (Fig. 1c, d). Gravid mosquitoes also demonstrated a dose-dependent attraction and oviposition preference to the EAK 71-402 headspace extract compared to the control solvent DCM (***χ***^2^ = 0.543, 95% CI 0.393–0.749, P < 0.001; (***χ***^2^ = 0.990, 95% CI 0.986–0.994, P < 0.0001; Fig. 1e, f). Unlike the response to the Coll 48 headspace extract, there was no loss of behavioural response to the EAK 71-402 headspace extract at the two highest release rates tested. There was no significant difference in the attraction or oviposition preference when controls were compared to controls alone, including DCM and DCM-conditioned water, respectively (***χ***^2^ = 1.094, 95% CI 0.798–1.499, P = 0.576; ***χ***^2^ = 1.001, 95% CI 0.995–1.006, P = 0.793; Fig. 1c–f). In a direct comparison between the headspace extracts of the two sugarcane cultivars, gravid *An. arabiensis* were more attracted to, and preferred to oviposit on, Coll 48 headspace extract at the three lowest release rates tested, while at the two highest release rates tested the EAK 71-402 headspace extract was more attractive and preferred for oviposition (Fig. 1g, h). This range of sensitivity of the mosquitoes was supported when compared to the response to the solvent in Fig. 1c, f. However, there was no overall significant difference in attraction (***χ***^2^ = 0.990, 95% CI 0.746–1.314, P = 0.946; Fig. 1g) and oviposition preference (***χ***^2^ = 1.003, 95% CI 0.760–1.325, P = 0.981; Fig. 1h) between the headspace volatiles of the cultivars.

### A complex blend of odours associated with sugarcane pollen drives mosquito oviposition behaviour

To identify the bioactive compounds associated with sugarcane pollen, the primary olfactory organ of gravid *An. arabiensis*, the antennae, were exposed to the pooled headspace volatiles of Coll 48 and EAK 71-402 sugarcane pollen, and the antennal response to compounds within the extracts was assessed using GC-EAD. The headspace extracts of both cultivars were pooled prior to GC-EAD to ensure that all bioactive compounds affecting the behaviour of the gravid mosquitoes were included in the screen. Compounds in the pooled extracts eliciting antennal responses were then identified (Fig. 1i) using GC–MS. The antennae responded consistently to 11 compounds in the sugarcane pollen-associated headspace extracts: *o*-xylene, styrene, (*1R*)-(+)-α-pinene, benzaldehyde, 1,8-cineole, nonanal, undecane, *p*-cymene, *N*-ethylbenzenamine, dibutyl phthalate and eicosane. These were confirmed to be bioactive following GC-EAD validation with authentic standards and used for behavioural assays (Fig. 1i). The total release rate of the compounds was ca. 175 ng min^−1^ (Fig. 1i). The most abundant compounds from the identified bioactive compounds were *o*-xylene and benzaldehyde.

**Fig. 2:**
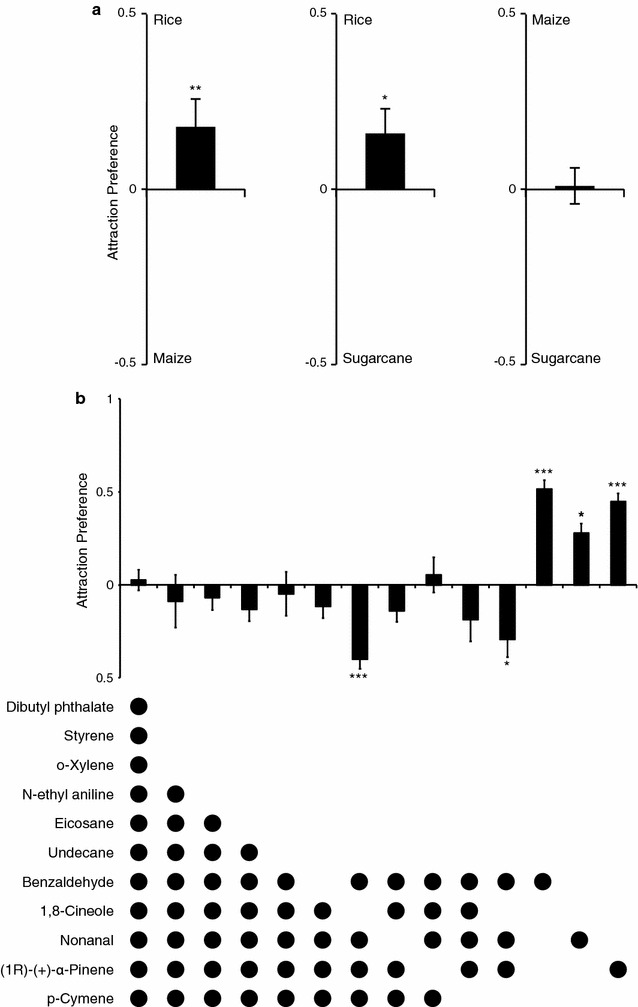
Attraction of gravid *Anopheles arabiensis* to grass-related volatile blends. Attraction of gravid females, in pairwise comparisons, to the synthetic rice plant, and maize and sugarcane pollen odour blends (**a**); and to the full synthetic (upper) and subtractive (lower) odour blends based on the sugarcane pollen (**b**). Ten replicates, of 10 mosquitoes each, were used in each behavioural experiment. Error bars represent standard errors of the mean. Asterisks indicate significant differences between the odour blends (*P < 0.05; **P < 0.01; ***P < 0.001)

The synthetic blend, consisting of the 11 commercially available identified sugarcane pollen associated volatiles, was made by mimicking the natural proportion of the compounds in the headspace extracts (Fig. 1i). Gravid *An. arabiensis* responded dose-dependently to the full synthetic blend in both the two-port olfactometer (***χ***^2^ = 0.648, 95% CI 0.484–0.868; P < 0.004; Fig. 1j) and oviposition bioassays (***χ***^2^ = 0.997, 95% CI 0.994–1.000; P < 0.024; Fig. 1k) compared with the solvent pentane. The release rate of the full blend that elicited the optimal behavioural response (50 ng min^−1^) was found to be similar to the natural release rate, 17 ng min^−1^, (*1R*)-(+)-α-pinene equivalents, that is within half an order of magnitude. Pairwise comparisons among the three identified synthetic blends for the domesticated grasses, revealed that rice was the most attractive blend for gravid *An. arabiensis* mosquitoes (rice vs. maize: ***χ***^2^ = 2.245, 95% CI 1.267–3.637; P < 0.005; rice vs. sugarcane: ***χ***^2^ = 0.588, 95% CI 0.378–0.912; P < 0.018; Fig. 2a). No significant difference in attraction was found between the maize and sugarcane pollen blends (***χ***^2^ = 0.961, 95% CI 0.552–1.672; P = 0.888; Fig. 2a). Subtractive assays revealed that benzaldehyde, nonanal and (*1R*)-(+)-α-pinene are key components of the synthetic blend, resulting in significantly stronger attraction in direct comparison with the full blend (***χ***^2^ = 4.502, P = 0.034; Fig. 2b). Addition of *p*-cymene to this subtractive blend, did not alter the significant attraction (***χ***^2^ = 23.907, P < 0.001; Fig. 2b). When each component was tested alone, the full blend was significantly more attractive to the gravid mosquitoes (benzaldehyde: ***χ***^2^ = 27.726, P < 0.001; nonanal: ***χ***^2^ = 5.026, P < 0.021; (*1R*)-(+)-α-pinene: ***χ***^2^ = 13.512, P < 0.001; Fig. 2b).

## Discussion

Volatiles associated with sugarcane pollen, like those with maize pollen [23], rice plants [24], and wild grasses [28] differentially attract and stimulate oviposition in gravid *An. arabiensis*, under laboratory conditions. As such, these odour cues provide a faithful signal for the gravid mosquitoes to locate and select the suitable larval habitats associated with wild and domesticated Poacae grasses [23, 24, 28]. Besides providing larvae with shelter from abiotic and biotic stresses [29], the pollen from Poacae grasses, particularly those rich in carbohydrates and nitrogen [15, 18, 19], provide a source of larval nutrition in an otherwise nitrogen resource limited environment [18, 30–32]. Larvae from Poacae grass pollen-rich environments show an enhanced development and survival [15, 18, 19], increasing the population density [10, 12]. The resulting adults display an increased vectorial capacity, which correlates with an increased risk of malaria in agricultural areas [8–12, 25]. Preferences for Poacae grasses and their volatiles, thus have a direct effect on vector dynamics, and provide a potential avenue for the manipulation of vector behaviour.

While *An. arabiensis* larvae can be found in a variety of habitats (e.g. [20]), they are frequently found in grass-associated habitats [1–7], which suggests a direct relationship between Poaceae, the true grasses, and the oviposition preference of malaria mosquitoes [10]. Cropping systems that include irrigated monocultures of sugarcane, maize and rice, provide suitable habitats for malaria mosquito larvae in proximity with human habitation [10, 12]. The human selection and continued cultivation of grass cultivars have inadvertently enhanced the nutritive qualities of the grass pollen, which provides a nutrient-rich resource for larvae [15, 18, 19]. The authors hypothesize that the fitness-related benefits of pollen in the mosquito diet [15, 18], may have exerted a selection pressure on the olfactory system of *An. arabiensis*.

While the composition of the synthetic sugarcane and maize pollen odour blends is unique [24, this study], the gravid females did not discriminate between these two blends. These blends share a common backbone of four compounds, (*1R*)-(+)-α-pinene, nonanal, *p*-cymene and benzaldehyde, and when tested against the full sugarcane odour blend, a blend of only these four compounds are found to be more attractive. The omission of *p*-cymene did not remove the attraction to this reduced blend, indicating a conserved olfactory mechanism in gravid malaria vectors for the identification and selection of grass pollen containing larval habitats. The context in which these compounds are presented, in terms of ratio, release rate and blend composition, however, has a direct effect on the behavioural output of the gravid mosquito [23, 24]. Pairwise comparisons between the identified synthetic rice odour blend [23] and the maize [24] as well as the sugarcane (this study) pollen odour blends, showed that the gravid mosquitoes preferred the rice odour blend. The composition of the rice odour blend, while sharing (*1R*)-(+)-α-pinene and nonanal with the pollen blends, does not contain *p*-cymene nor benzaldehyde, but contains another shared compound with maize pollen, (*R*)-(+)-limonene. While the release rates and relative ratios of the shared compounds within the blends differ [23, 24, this study], a combination of these compounds in various ratios and release rates may lead to the development of a chimeric blend, which could have an increased capacity to attract gravid malaria mosquitoes compared to the synthetic blends based on the natural odours.

Synthetic odour blends have been gaining ground as tools for the manipulation of disease vector behaviour in vector monitoring and control programmes [33, 34]. Existing odour lures, including complex blends rather than single components, have been used with some success to target predominantly indoor blood host-seeking females [33, 34]. The identification of volatile blends from oviposition sites have the advantage of attracting gravid mosquitoes, regardless of where these females have rested and fed. Hence, a gravid lure derived from attractive grasses, when integrated into other control tools, has the advantage of increased monitoring accuracy and likelihood of a representative capture rate of the targeted vector species, by providing better estimates of the number of egg-laying females, as well as the potential of a more dynamic control of mosquito populations regardless of the vectors’ insecticide or behavioural resistance. Ongoing landscape analyses will provide concerted information about the most effective locations for the placement of traps and monitoring devices within and around rural communities. From an integrated vector management perspective, the use of an effective chimeric, rather than a natural, odour blend increases the potential to avoid unnecessary competition with the natural odours within the environment.

## Conclusions

Gravid *An. arabiensis* were attracted to blend volatile emitting from potential oviposition site of grass maize pollen and rice plants, from present study to sugarcane pollen. Of these, rice plant volatiles were more preferred by gravid mosquitoes while maize and sugarcane pollens volatiles were equally attractive. Using the synthetic blends from the three grasses, we are currently developing a chimeric blend, to be evaluated under laboratory and field conditions. The outcomes from that study will increase the control options for outdoor mosquitoes and may be used in combination with other control options.

## Abbreviations

AP: attraction preference
C: control
CI: confidence interval
CAS no.: chemical abstracts service number
***χ***^2^: chi squared
DCM: dichloromethane
EAD: electroantennogram detection
FB: full blend
FID: flame ionization detector
GC: gas chromatography
PEN: pentane
MS: mass spectrometry
OP: oviposition preference
T: test
